# When Do We Confuse Self and Other in Action Memory? Reduced False Memories of Self-Performance after Observing Actions by an Out-Group vs. In-Group Actor

**DOI:** 10.3389/fpsyg.2012.00467

**Published:** 2012-11-02

**Authors:** Isabel Lindner, Cécile Schain, René Kopietz, Gerald Echterhoff

**Affiliations:** ^1^Cognitive Psychology Group, Department of Psychology, University of KasselKassel, Germany; ^2^Social Psychology Group, Department of Psychology, University of MünsterMünster, Germany

**Keywords:** self-other confusion, group membership, action memory, false memory, motor simulation, perceptual distinctiveness

## Abstract

Observing another person performing an action can lead to a false memory of having performed the action oneself – the observation-inflation effect. In the experimental paradigm, participants first perform or do not perform simple actions, and then observe another person perform some of these actions. The observation-inflation effect is found when participants later remember performing actions that they have merely observed. In this case, self and other are confused in action memory. We examined social conditions of this self-other confusion when remembering actions, specifically whether the effect depends on the observed actor’s group membership. In our experiment, we manipulated group membership based on physical appearance, specifically complexion of the hands. Fair-skinned participants observed either an in-group (i.e., fair-skinned) or an out-group (i.e., dark-skinned) actor. Our results revealed that the observed actor’s group membership moderated the observation-inflation effect: False memories were significantly reduced when the actor was from the out-group (vs. in-group). We found no difference to a control condition in which the actor wore black gloves, suggesting that distinctiveness of perceptual or sensory features alone (due to the out-group member’s dark skin) is not critical. We discuss these findings in light of social-neuroscience studies demonstrating the impact of an observed person’s group membership on motor simulation. Overall, our findings suggest that action memory can be affected by a ubiquitous feature of people’s social perception, that is, group-based social categorization of others.

## Introduction

Imagine that you watch a cooking-show on television while you are preparing dinner yourself. Sometime later, after you have left the house, you find yourself wondering whether you actually switched off the stove. Clearly, correctly remembering whether one has performed an action is crucial for successfully managing everyday life. Did you just observe the TV-cook switching off his or her stove, or did you do it yourself? Recent research shows that observing another person’s action can lead people to mistakenly remember that they have performed the action themselves (Lindner et al., 2010; Schain et al., 2012). The present research was conducted to investigate conditions under which such false self-attributions of actions are more or less likely to occur. We focused on social conditions, specifically the observed actor’s membership in the observer’s in-group (vs. out-group).

In the initial experimental demonstration of the effect, Lindner et al. (2010) asked participants to perform or to read simple action statements, like “*Unlock the lock*” or “*Shake the bottle*.” Afterwards, participants were asked to observe another person perform some of the actions they had and some of the actions they had not previously performed themselves. Two weeks later, a surprise source-memory test revealed that observation of other-performed actions had inflated false memories of self-performance. Thus, this effect has been referred to as *observation inflation* (see also *imagination inflation*, i.e., false memories of self-performance from imagination; Garry et al., 1996; Goff and Roediger, 1998).

The observation-inflation effect reveals the profoundly social nature of memory inasmuch as it entails a confusion of self and other in recollecting actions. Thus, this phenomenon is a prime example of how our contact with the social world affects our memory (see Echterhoff and Hirst, 2009; Hirst and Echterhoff, 2012). Because we constantly observe others performing actions, there are countless occasions that potentially could trigger subsequent false self-attributions of actions. But do we indiscriminately incorporate others’ actions into our own action memories? Or do such self-other confusions depend on social characteristics of the observed actor?

Research on social perception and interpersonal processes suggests that we are more likely to adopt, or to be affected by, the experiences of others who are more (vs. less) close and similar to ourselves (Aron et al., 2004). A key component involved in feelings of closeness and similarity is another person’s group membership. One of the first things we do when we are observing another person – often automatically and spontaneously – is to categorize her or him (Macrae and Bodenhausen, 2000). Using cues like a person’s age, gender, and race we sort people into different social groups that are either like us (i.e., the in-group) or not like us (i.e., the out-group).

Regarding the present phenomenon, there is ample evidence that people more readily adopt and incorporate the inner states of in-group (vs. out-group) members (Echterhoff et al., 2009; Cikara et al., 2011). This bias has been found for affects and emotions (Xu et al., 2009; Avenanti et al., 2010; Azevedo et al., in press), but also for memory (Echterhoff et al., 2008, in press).

So far, only few studies have examined whether and how memory for another person’s features and actions is influenced by the person’s group membership. For example, people are better in remembering in-group than out-group faces (see Meissner and Brigham, 2001; Young et al., 2012), and memory for negative out-group behavior is better than for positive out-group behavior (Howard and Rothbart, 1980). Furthermore, people are more likely to confuse the sources of recalled statements when the sources belong to the same (vs. a different) ethnic or racial group (Taylor et al., 1978; Stangor et al., 1992). With the present research we specifically examined whether false action memories from observation also depend on the observed others’ group membership.

While the impact of group membership on action memory in general and false action memory in particular has not been investigated, there are potentially relevant findings outside the memory literature. Studies on action perception hint at a consistent pattern between observation of others’ actions and their group membership. In one study, Müller et al. (2011) employed the social Simon task (Sebanz et al., 2003) to measure coordinated action between interaction partners. It was found that participants’ reaction times were decelerated when interacting with an out-group (vs. in-group) member, indicating that co-representations of actions are only formed when coordinating with in-group members. Similarly, people exhibit increased cortical sensitivity when observing errors in action execution of someone who is similar (vs. dissimilar; Carp et al., 2009; Newman-Norlund et al., 2009; Kang et al., 2010). Furthermore, Gutsell and Inzlicht (2010) found that motor simulation during action observation was substantially decreased when observing out-group (vs. in-group) members. Taken together, actions of out-group members tend to be processed less preferentially and represented less readily in the observer than actions of in-group members. Accordingly, the potential of observed actions to induce false action memories in the observer should be less pronounced after observation of an out-group (vs. in-group) member.

The present study was designed to test this prediction. We manipulated a person’s group membership by presenting either a fair-skinned (in-group) or a dark-skinned (out-group) actor to fair-skinned observers and predicted that a self-other confusion in action memory should be accentuated after observation of an in-group member, but reduced after observation of an out-group member.

A reduced observation-inflation effect after observation of an out-group member could be due to mere perceptual distinctiveness of the dark-skinned hands. To control for this possibility, participants in a control group observed an actor wearing black gloves (see Avenanti et al., 2010; Azevedo et al., in press). Whereas an approach drawing on perceptual distinctiveness predicts the least false memories after observation of a person wearing black gloves, an intergroup-bias approach suggests a reduced observation-inflation effect exclusively after observing an out-group member, but not after observing a person wearing black gloves.

## Materials and Methods

### Participants

Fifty-eight students at the University of Münster (mean age = 21.9, *SD* = 4.7, 11 men) participated in this study for partial course credit. All participants were fair-skinned. The study was approved by the local ethics committee. The guidelines of the Declaration of Helsinki and standards of the American Psychological Association were followed. Informed consent was obtained from all participants.

### Design

A 2 (observation, Phase 2: observed vs. not observed) × 3 (group membership of observed actor, Phase 2: in-group vs. out-group vs. control) design was used, with the first variable manipulated within participants. The main dependent measure was the relative frequency of false *performed*-responses for actions that were only read but not performed in Phase 1.

### Materials and procedure

We adapted the procedure introduced by Lindner et al. (2010). The experiment was computer-based and consisted of two sessions, separated by a 2-week interval. Participants were tested individually in both sessions.

In Phase 1, for each participant 40 action statements were randomly chosen from a pool of 60 action statements and presented at the center of a 22-inch TFT-LCD display in a random order. Half of the action statements had to be performed, the other half were only read. Assignment of items to these two types of encoding was also randomized. At the beginning of each trial, the name of a specific object (e.g., *Lock*) appeared on the screen. Objects were hidden behind a visual cover, thus participants waited until the experimenter had placed the required object in front of them. Next, a perform instruction (*Please perform*) or a read instruction (*Please read*) appeared on the screen and was followed by the specific action statement (e.g., *Unlock the lock!*). Participants now performed or read out the action statement once before moving on to the next trial.

In Phase 2, half of the action statements performed in Phase 1 and half of the action statements read in Phase 1 were randomly chosen and presented. Each presentation of an action statement was followed by a corresponding video showing the specific action repeatedly over the course of 15 s. To maintain participants’ attention, an observation task was introduced: Participants were asked to count the number of action repetitions after each video and enter their final count. Overall, each action statement and the corresponding video were presented five times in a random order, resulting in a total of 100 trials for Phase 2.

**Figure 1:**
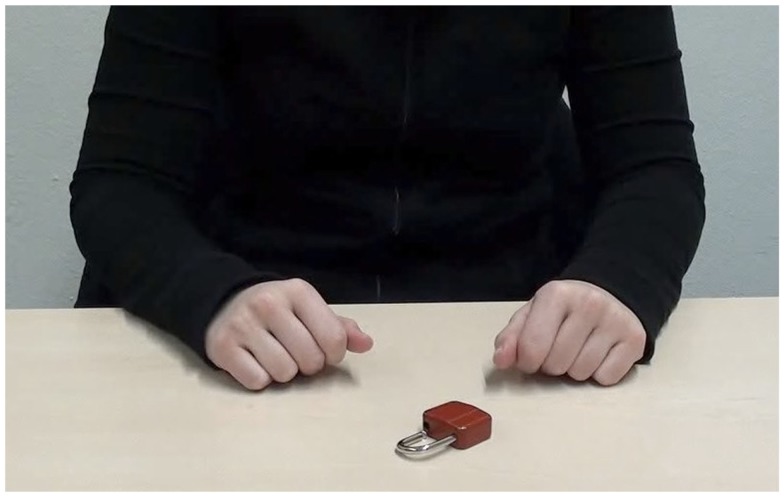
**Screenshot of action video *Unlock the lock!* presented in the *in-group* condition**.

**Figure 2:**
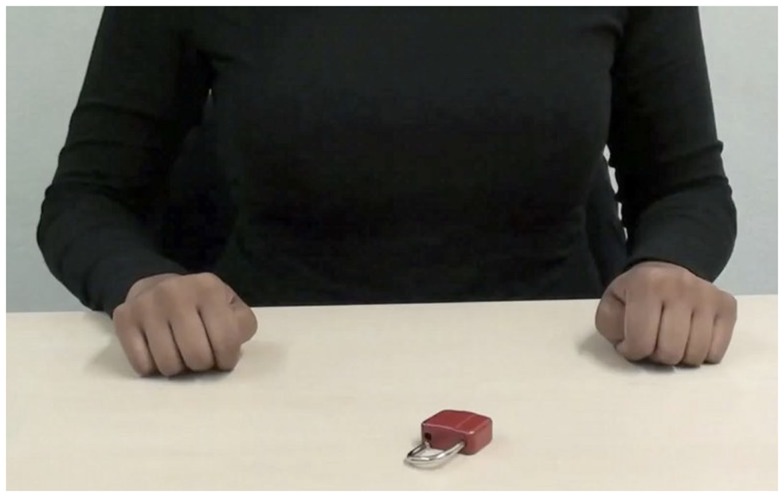
**Screenshot of action video *Unlock the lock!* presented in the *out-group* condition**.

**Figure 3:**
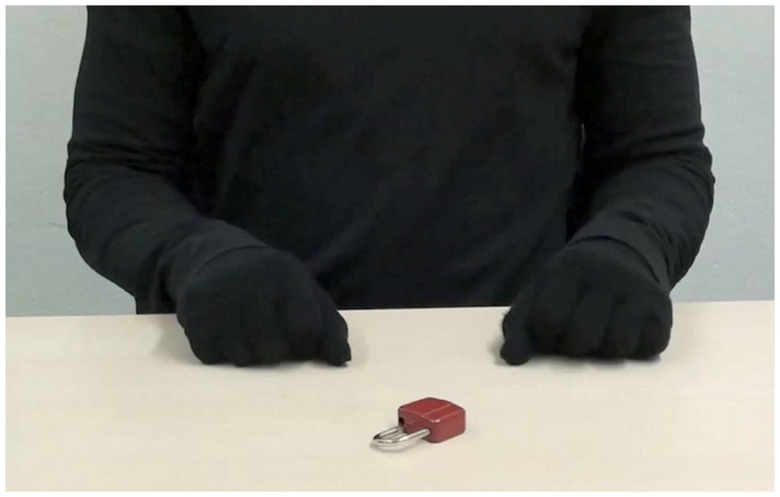
**Screenshot of action video *Unlock the lock!* presented in the *control* condition**.

In the *in-group* condition, videos of the torso, forearms, and hands of a German, fair-skinned female actress (24 years) were presented from a second-person perspective (352 kb/s, 960 × 530 pixels, see Figure 1). In the *out-group* condition, actions were performed by a Sri Lankan, dark-skinned female actor (21 years, see Figure 2). A pretest (*N* = 17 women; mean age = 22.18; *SD* = 2.19) confirmed that the different ethnicities could be easily derived from the videos. Participants rated sample videos with regard to the estimated likability for several countries and regions on a seven-point Likert scale (one being *very unlikely*, seven being *very likely*). Compared to the in-group actress, the out-group actress was perceived as significantly less likely to be from Germany [out-group: *M* = 3.1, *SD* = 1.8; in-group: *M* = 6.2, *SD* = 1.1; *t*(16) = 5.83, *p* < 0.001, *d* = 1.41], and more likely to be from South Asia [out-group: *M* = 5.1, *SD* = 1.8; in-group: *M* = 2.5, *SD* = 1.2; *t*(16) = 6.75, *p* < 0.001, *d* = 1.64]. For the *control* condition, the actor (22 years) wore black gloves during action performance. The actor’s actual skin color (fair) was not visible at any time (see Figure 3).

Two weeks later, the 40 old action statements were randomly presented in a surprise source-memory test along with 20 new action statements. Participants indicated for each action statement whether it was performed, read, or not presented during Phase 1.

Our hypotheses refer to false *performed*-responses, that is, *performed*-responses to actions only read in Phase 1. An observation-inflation effect was defined as a significant increase in false *performed*-responses for items observed compared to items not observed in Phase 2. Accordingly, the magnitude of the effect was defined as the difference in false *performed*-responses between items observed and not observed in Phase 2.

## Results

To test our hypotheses, we ran planned pairwise comparisons (Type I error threshold = 0.05). Accordingly, *p*-values are one-tailed.

Table 1 contains the means and standard deviations of false *performed*-responses for the three experimental conditions (in-group actor vs. out-group actor vs. control). We found significant observation-inflation effects in all three conditions, the in-group condition, *t*(19) = 6.40, *p* < 0.001, *d* = 1.43, the out-group condition, *t*(18) = 6.32, *p* < 0.001, *d* = 1.44, and the control condition, *t*(18) = 4.17, *p* < 0.001, *d* = 0.96, respectively. Thus, all participants were prone to falsely claiming that they had performed actions themselves when in fact they had only observed another person performing these actions.

**Table 1:** **Mean proportion of performed-responses as a function of encoding in Phase 1, observation in Phase 2, and observation group**.

Encoding, Phase 1	Observation, Phase 2					
	In-group		Out-group		Control: black gloves	
	Observed	Not observed	Observed	Not observed	Observed	Not observed
Performed	0.79 (0.17)	0.46 (0.15)	0.80 (0.14)	0.56 (0.23)	0.72 (0.16)	0.58 (0.21)
Read	0.27 (0.18)	0.06 (0.08)	0.18 (0.15)	0.05 (0.10)	0.24 (0.17)	0.08 (0.10)
Not presented	–	0.02 (0.04)	–	0.01 (0.02)	–	0.01 (0.03)

*Standard deviations are given in parentheses. Proportions represent the frequency of performed-responses divided by the number of all responses for a corresponding item type*.

**Figure 4:**
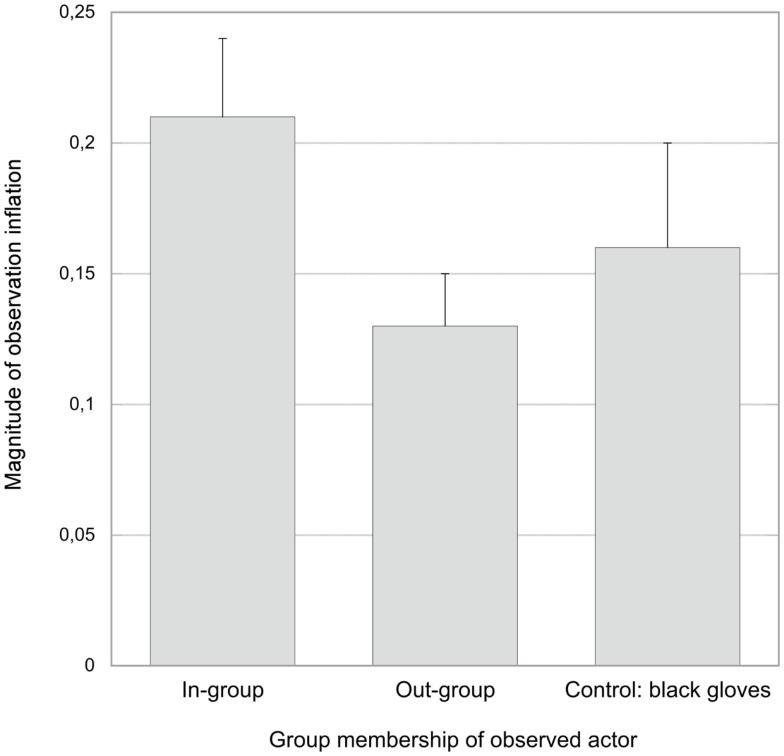
**Magnitude of the observation-inflation effect as a function of group membership of the observed actor**. Error bars represent the standard error of mean.

Importantly, the magnitude of the effect, that is, the difference in false *performed*-responses between observed and not observed items, was a function of the actor’s group membership (see Figure 4). As predicted, planned comparisons indicated that the effect was significantly lower in the out-group than in the in-group condition, *t*(37) = 2.06, *p* = 0.02, *d* = 0.65. No significant difference was found between the control condition and the in-group condition, *t*(37) = 0.83, *p* = 0.21, *d* = 0.27, or between the control condition and the out-group condition, *t*(36) = 0.84, *p* = 0.20, *d* = 0.27.

We also conducted additional analyses to examine the role of other alternative processes (differences in task motivation, attention, or a general response bias) that could account for the present findings. First, we analyzed performance on the counting task that was administered in Phase 2 (that is, the counting of observed action repetitions). Mean accuracy rates varied between 0.79 and 0.85, and did not differ between the three experimental conditions, *F*(2, 51) = 1.34, *p* = 0.27, η*_p_*^2^ = 0.05. This finding is inconsistent with the notion that the reduced observation-inflation effect in the out-group condition is due to decreased task motivation or visual attention, which could impair source memory in general.

Furthermore, the rate of false *performed*-responses to completely new items (not presented in Phase 1 and not observed in Phase 2) was close to zero, ranging from 0.01 to 0.02 in all the three experimental groups. Thus, differences in a general response bias toward claiming actions as self-performed cannot account for the difference in observation inflation found in the earlier main analysis.

## Discussion

Observation of another person performing a simple action can lead to a false memory of having performed this action oneself, that is, the observation-inflation effect (Lindner et al., 2010). The present study extends this initial finding inasmuch as it shows that the ethnic group membership of the observed person – conveyed by mere skin color – has an impact on the magnitude of the observation-inflation effect. Compared to an in-group (i.e., a fair-skinned) actor, the observation of an out-group (i.e., a dark-skinned) actor led to a significant reduction in false self-attributions of action performance after two weeks. As predicted from a social-psychological account of intergroup bias, such a reduction was not found for the observation of an actor wearing black gloves.

Our findings resonate well with research on reduced motor simulation of actions of out-group (vs. in-group) members. Building on research on mirror-neuron activity and shared motor representations, Lindner et al. (2010) hypothesized that motor simulation during action observation might be the core mechanism behind the observation-inflation effect. Apparently, people do not co-represent or share out-group members’ actions to the same extent than in-group members’ actions. Indeed, an EEG-study by Gutsell and Inzlicht (2010) suggests that covert vicarious action performance depends on an observed actor’s group membership. These authors asked fair-skinned Canadians to either perform a target action themselves or to observe an in-group member (Caucasian) vs. an out-group member (African-Canadian, East-, or South-Asian) performing this same action. In these different conditions, they measured suppression of EEG activity in the mu frequency bandwidth over the primary motor cortex, which is thought to index the degree of motor simulation. Gutsell and Inzlicht found that both, performing an action oneself and observing an in-group member performing an action, elicited mu suppression. However, there was no significant mu suppression, that is, engagement in motor simulation, when observing an out-group member.

Furthermore, our results for the black-glove control condition are consistent with findings by Avenanti et al. (2010). These authors asked fair- and dark-skinned participants to observe the hands of either a fair-skinned, a dark-skinned, or an (artificially) violet-skinned actor experiencing pain (vs. no pain). The findings revealed sensorimotor resonance, indicating neuronal simulation of the observed pain, for observation of the in-group member, but not of the out-group member. They also found that observers simulated the pain of the violet-skinned actor, but to a smaller extent than for the in-group member. Analogous to this finding, in our study, the observers might have felt uncertain about the group membership of the person wearing black gloves. Hence, the observation of the glove-wearing actor would elicit only an intermediate level of motor simulation.

How can biased motor simulation account for the present memory effects? The bulk of the motor simulation literature, including studies on intergroup differences (Xu et al., 2009; Avenanti et al., 2010; Gutsell and Inzlicht, 2010; Azevedo et al., in press), has focused on the processes ongoing *during* observation. However, a few studies have shown that motor representations from observation are reactivated during retrieval (Senkfor et al., 2002; Wutte et al., 2012). Consistent with a motor-simulation account of the present false memory effect, Wutte et al. (2012) found overlapping neural activation in motor areas when participants remembered self-performed and observed movements.

Our findings extend the common understanding of the emergence of false memories of self-performance. Explanations of related false action memories, specifically the imagination-inflation effect (Goff and Roediger, 1998), have invoked the misattribution due to similarity of sensory features. According to this account, which draws on the source-monitoring framework (Johnson et al., 1993), non-self-performed actions (e.g., merely imagined actions) are attributed to self-performance to the extent that the sensory features of action memories from the competing sources are similar (vs. dissimilar). For instance, after vividly imagining the action of shaking a bottle, people may later remember perceptual details and features, such as shape and color of the bottle, or fingers grasping the bottle; because these features are similar to the features contained in memories of self-performed actions, an originally imagined action may be misattributed to previous self-performance. Conversely, by this view, the availability of distinctive perceptual features at retrieval prevents such misattributions.

Our findings are not easily reconciled with this account. Because hands wearing black gloves are more salient and perceptually more distinct from fair-skinned hands than dark-skinned hands, the sensory-feature account of misattribution would predict that this condition results in the lowest rate of false action memories. However, observation inflation was lowest in the out-group condition, indicating that group membership rather than perceptual distinctiveness was critical in forming false memories of self-performance. This result is in line with earlier findings (Lindner et al., 2010, Exp. 3).

Still, whereas similarity of perceptual features is not sufficient to account for the pattern of results that we found in the current study, the distinctiveness of such features may still moderate the effect under certain circumstances. Schain et al. (2012) manipulated perceptual distinctiveness by using the actor’s face as a visual identity cue. They found that observation inflation is diminished when a central identity cue, that is, the actor’s face, was available and attended to by observers. In light of these previous findings, one might wonder why the non-self cues employed here, dark skin or black gloves, did not eliminate false self-attributions to a greater extent. It could allow observers to apply a rule such as “*I did not wear black gloves/my skin is not that dark, so it was not me who performed this action*” to avoid false self-attributions.

This issue can be resolved by arguing that the face is probably a more effective and distinctive identity cue than are skin color or a piece of clothing. The face offers various features (e.g., color of the eyes, size of the nose, shape of the forehead, etc.), which collectively signal a *Not-me*-response. Also, the detection and identification of others’ faces are exceptionally important in social development and social cognition (Zebrowitz, 1997), and humans, including newborns, have a pronounced tendency to direct their visual attention to others’ faces (Johnson and Morton, 1991). However, for engagement and modulation of motor simulation to occur, skin color has not to be highly salient – the observers’ categorization into an in-group vs. an out-group member is sufficient to alter shared motor representations in the observer.

To conclude, in the present study, we focused on physical similarity in terms of skin color to manipulate group membership. Future studies should examine whether our results generalize to other cues of group membership that are not conveyed by physical appearance and to other types of group membership than ethnicity. For instance, would we find the same bias when ethnicity is conveyed solely by verbal labels, such as first names that are typical for specific groups? And will observation inflation be higher if the actor shares, for example, the same values, the same profession, or the same interest as the observer?

With this study we have shown that people are less prone to confuse self and other in action memory when they have observed an out-group (vs. in-group) member. As we have argued, our findings are not easily explained by the similarity or distinctiveness of perceptual features, but are consistent with an account of intergroup biases in motor simulation. Future studies should seek further evidence for the underlying mechanisms in this fascinating new domain of memory research.

## Conflict of Interest Statement

The authors declare that the research was conducted in the absence of any commercial or financial relationships that could be construed as a potential conflict of interest.
